# Construction of Vero cell-adapted rabies vaccine strain by five amino acid substitutions in HEP-Flury strain

**DOI:** 10.1038/s41598-024-63337-9

**Published:** 2024-05-31

**Authors:** Michiko Harada, Aya Matsuu, Eun-Sil Park, Yusuke Inoue, Akihiko Uda, Yoshihiro Kaku, Akiko Okutani, Guillermo Posadas-Herrera, Keita Ishijima, Satoshi Inoue, Ken Maeda

**Affiliations:** 1 Joint Graduate School of Veterinary Medicine, Yamaguchi University, 1677-1 Yoshida, Yamaguchi, 753-8515 Japan; 2 Department of Veterinary Science, National Institute of Infectious Diseases, 1-23-1 Toyama, Shinjuku-ku, Tokyo, 162-8640 Japan

**Keywords:** Vaccines, Virology

## Abstract

Rabies virus (RABV) causes fatal neurological disease. Pre-exposure prophylaxis (PrEP) and post-exposure prophylaxis (PEP) using inactivated-virus vaccines are the most effective measures to prevent rabies. In Japan, HEP-Flury, the viral strain, used as a human rabies vaccine, has historically been propagated in primary fibroblast cells derived from chicken embryos. In the present study, to reduce the cost and labor of vaccine production, we sought to adapt the original HEP-Flury (HEP) to Vero cells. HEP was repeatedly passaged in Vero cells to generate ten- (HEP-10V) and thirty-passaged (HEP-30V) strains. Both HEP-10V and HEP-30V grew significantly better than HEP in Vero cells, with virulence and antigenicity similar to HEP. Comparison of the complete genomes with HEP revealed three non-synonymous mutations in HEP-10V and four additional non-synonymous mutations in HEP-30V. Comparison among 18 recombinant HEP strains constructed by reverse genetics and vesicular stomatitis viruses pseudotyped with RABV glycoproteins indicated that the substitution P(L115H) in the phosphoprotein and G(S15R) in the glycoprotein improved viral propagation in HEP-10V, while in HEP-30V, G(V164E), G(L183P), and G(A286V) in the glycoprotein enhanced entry into Vero cells. The obtained recombinant RABV strain, rHEP-PG4 strain, with these five substitutions, is a strong candidate for production of human rabies vaccine.

## Introduction

Rabies is a lethal zoonotic disease that induces encephalitis in almost all species of mammals; the disease has been reported around the world, and is especially prevalent in Asia and Africa^1^. Globally, the annual number of human deaths is estimated to be 59,000^2^, and more than 99% of human rabies cases are transmitted by dogs^3^. The World Health Organization (WHO) is promoting “the global strategic plan to end human deaths from dog-mediated rabies by 2030: Zero by 30”^4^. Rabies vaccine for dogs have been recommended around the world^5,6^, and the implementation of vaccine programs in each region has contributed to a reduction in the frequency of dog rabies, in turn resulting in a decrease in the number of human rabies cases^1^.

The rabies virus (RABV) is a non-segmented, single stranded, negative-sense RNA virus that belongs to the order Mononegavirales, family *Rhabdoviridae*, subfamily *Alpharhabdovirinae*, genus *Lyssavirus*^7^. The virus encodes 5 proteins, including the nucleoprotein (N protein), phosphoprotein (P protein), matrix (M) protein, glycoprotein (G protein) and a large RNA-dependent RNA polymerase (L protein)^1,3,8–11^. The N protein forms a ribonucleoprotein (RNP) complex with the P and L proteins; the resulting complex promotes mRNA production as well as full-length genomic RNA replication^8,9^. The P protein has multiple functions, including the regulation of replication and transcription, facilitation of axonal transport, and inhibition of interferon (IFN) production by the host^9,11,12^. The M protein induces the assembly and budding of novel viral particles^9^. The G protein is associated with binding to the cell receptor, induction of virus-neutralization antibodies (VNAs), and pathogenicity in humans and animals^3,8–10,13^. The L protein, in combination with the P protein, is involved in the replication and transcription of the viral genome, as well as in evasion of host innate immunity^9,13^.

After an incubation period of 1–3 months, RABV causes neurological signs in humans; there are no effective treatments once the symptoms develop. However, post-exposure prophylaxis (PEP) using rabies vaccine and anti-rabies immunoglobulins, permits survival of infected individuals without clinical symptoms, if initiated soon after exposure^1,14,15^. In addition, pre-exposure prophylaxis (PrEP) using rabies vaccine also is available for people with occupations engendering a high risk of rabies infection or who travel to areas where rabies is endemic^1,15,16^.

The Flury strain of RABV, which was isolated from a girl who died of rabies, was adapted to growth in 1-day-old chicks in 1940^17^. Subsequently, the Flury strain was passaged 40–50 times in chicken eggs to generate the low-egg-passage Flury strain (LEP-Flury)^17–19^. The original Flury strain also was passaged 180 times or more in eggs to generate the high-egg-passage Flury strain (HEP-Flury)^17–20^. LEP-Flury has pathogenicity in adult mice, while HEP-Flury is lethal only in suckling mice, and not in adult mice^19,21,22^. The purified chick embryo cell vaccine (PCECV) was first produced from the HEP-Flury strain in 1972 and has been manufactured in Japan since 1980^14,17,18^. Another PCECV developed from the LEP-Flury strain is used in Europe and the USA^17^. In Japan, the chick embryo cell-adapted HEP-Flury small plaque-forming (CEF-S) strain, which was generated by further passages of HEP-Flury in primary chick embryo cells, has been used to produce PCECV^18^. In Japan, the Rabipur rabies vaccine (GSK Biologicals, Wavre, Belgium) has been licensed for use since 2019. This vaccine, a PCECV derived from LEP-Flury, is imported for use in Japan^17,23^. However, there are problems in the production of vaccine using primary chicken embryo cells, because preparation of those cells require special techniques and processing time and high costs.

In 1962, the Vero cell line was established from African green monkey kidney cells^24^. In the late 1970s and early 1980s^17^, an inactivated polio vaccine was produced using the Vero cell line^25,26^. Subsequently, Vero cell-cultured rabies vaccines were developed in the early 1980s; one such purified Vero cell rabies vaccine (PVRV) has been used in Europe since 1985^9,17,27^. Among the PVRVs, one is produced using the PV-2061 rabies strain, a virus that has been adapted for growth in Vero cells^14^. The PV-2061 strain was generated by passage of the Louis Pasteur strain (PV)^28,29^; passaged 2061 times in rabbit brain, 19 times in the Vero cell line, and five times in baby hamster kidney (BHK) cells^17^. The Verorab rabies vaccine (Sanofi Pasteur, Lyon, France), which is produced using PV-2061^17,23,30^, and is used world-wide as a PVRV for humans, but this vaccine is not licensed in Japan.

The HEP-Flury strain replicates to high titers in mouse neuroblastoma (MNA) cells as well as neuronal cells^13,21^, but not in Vero cells^31^. However, the WHO recommends against the use of neuronal cells for the development of human rabies vaccines^1^, given that vaccines generated is such cells may induce, in humans, side effects such as acute disseminated encephalomyelitis^1,32^. In Japan, vaccines against Japanese encephalitis^33,34^ and polio^26^ that were produced in Vero cells have been licensed. In this study, the HEP-Flury strain was adapted to Vero cells to facilitate effective vaccine production, and the functions of amino acid (AA) substitutions induced by adaptation to the Vero cell line were analyzed. Finally, the recombinant virus containing five AA substitutions was produced by reverse genetics as a novel vaccine seed for production of PVRV.

## Results

### Adaptation of HEP-Flury to Vero cells

Before adaptation of our HEP-Flury (HEP) to Vero cells, we determined the complete genome sequence of the HEP strain used in our laboratory and compared that sequence to those reported for other HEP-Flury strains (Supplemental Table S1). The genome of our strain was most similar to that previously reported^35^ as HEP-Flury strain Accession Number AB085828, and had only one AA substitution in the signal peptide of G protein. The CEF-S strain (Accession No. LC717412), which is propagated in chick embryo fibroblast cells and is used as the Japanese vaccine strain, had twenty AA substitutions compared to our HEP: four substitutions in the P protein, three substitutions in the M protein, nine substitutions in the G protein and four substitutions in the L protein.

In the present study, we sought to adapt our HEP strain to Vero cells to facilitate efficient vaccine production in this cell line. To this end, HEP was subjected to 30 rounds of serial passages in Vero cells. At passages 1 and 2, the infected Vero cells were blindly passaged because cytopathic effect (CPE) was not observed. At passage 3, CPE was observed at 6 days post infection (d.p.i.) and all subsequent viral passages consisted inoculation of the culture supernatants into Vero cells. From passages 4 to 17, HEP caused CPE at 3 d.p.i., and from passage 18, CPE was observed at 2 d.p.i.

### Comparison of viral growth of HEP and adapted HEPs in Vero cells

**Figure 1:**
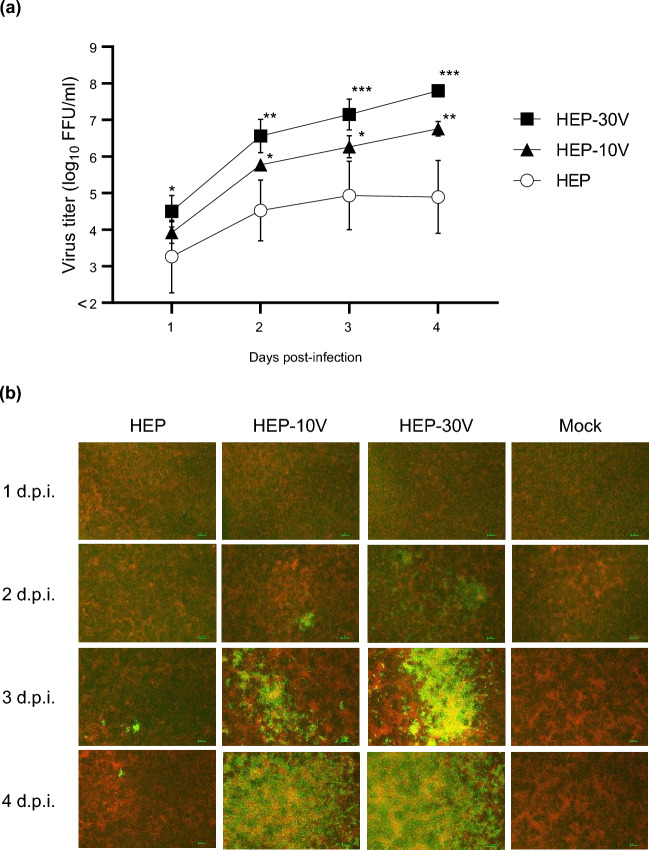
Viral growth and spread of HEP-Flury (HEP) and Vero-adapted viruses (HEP-10V and -30V) in Vero cells. Vero cells were inoculated with HEP, HEV-10V, or HEV-30V at a multiplicity of infection (M.O.I.) of 0.05. Supernatants were collected once daily through 4 days post infection (d.p.i.). (**a**) Viral titers were determined using MNA cells. Antigen-positive foci were counted under a fluorescence microscope and quantified as focus forming unit (FFU) per milliliter. Viral titers are plotted as the mean and standard deviation (S.D.) from three independent experiments. Significant differences are indicated (*: *p* < 0.05, **: *p* < 0.01, ***: *p* < 0.001) between HEP and HEP-10V or HEP-30V after application of two-way analysis of variance (ANOVA) followed by Tukey. HEP-10V vs HEP-30V had no significant difference at all time points (*p* > 0.05). (**b**) At the indicated time points, virus- and mock-infected cells were fixed with 80% cold acetone. Fixed cells were stained with the fluorescein isothiocyanate (FITC) anti-rabies monoclonal globulin (FUJIREBIO, Tokyo, Japan) and examined under a fluorescence microscope. The stained cells were observed using NIS-Elements D version 5.20.00 imaging software (Nikon, Tokyo, Japan). Cells are stained red with Evans Blue (FUJIFILM Wako Pure Chemical Corporation, Osaka, Japan). Scale bars, 100 μm; magnification, ×40.

To compare viral growth among the adapted HEP strains, Vero cells were infected at multiplicity of infection (M.O.I.) of 0.05 with HEP or the adapted strains that had been passaged 10 and 30 times in Vero cells, designated HEP-10V and HEP-30V, respectively (Fig. 1a and Supplemental Table S2). HEP-10V and HEP-30V accumulated to significantly higher titers than HEP did when grown in Vero cells, as assessed at 1–4 d.p.i. (HEP-10V; *p* = 0.046, 0.031, 0.002, HEP-30V; *p* = 0.047, 0.001, < 0.001, < 0.001, respectively). Additionally, HEP-30V accumulated to titers that were nominally higher than those of HEP-10V when grown in Vero cells without statistical significance.

To visualize differences in viral spread when propagated in Vero cells, HEP, HEP-10V, and HEP-30V were infected Vero cells at an M.O.I. of 0.01, and the infected cells were fixed and stained with fluorescein isothiocyanate (FITC)-conjugated anti-RABV antibody at 1–4 d.p.i. (Fig. 1b). For those cells, RABV-positive cells were observed at 2 d.p.i. However, the number of infected cells increased more rapidly in HEP-10V- and HEP-30V-infected cells than in HEP-infected cells, and the number of infected cells increased more rapidly in HEP-30V-infected cells than in HEP-10V-infected cells.

### Comparison of expression of viral proteins by HEP and the adapted HEPs

**Figure 2:**
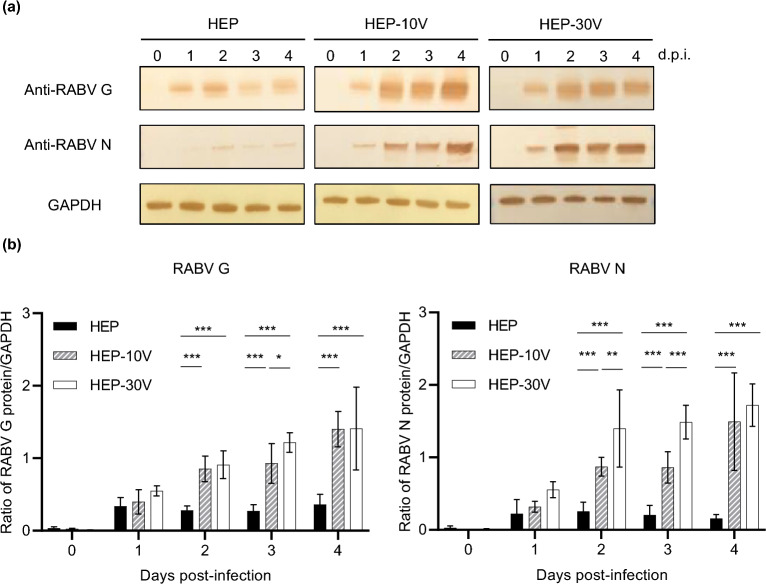
Comparison of expression of viral proteins among HEP, HEP-10V and HEP-30V in Vero cells. Vero cells were inoculated with each virus at an M.O.I. of 5 and harvested every day. (**a**) Rabies virus (RABV) glycoprotein (G protein) and nucleoprotein (N proteins) were visualized using polyclonal rabbit anti-serum against each protein. Glyceraldehyde-3-phosphate dehydrogenase (GAPDH), a housekeeping protein, was detected by monoclonal mouse antibody for use as a loading control. (**b**) Virus-specific bands were quantified using ImageJ software (National Institutes of Health, Bethesda, MD, USA) and levels of RABV G and N proteins were normalized to those of GAPDH. The means and S.D. were calculated from two independent experiments. Significant differences are indicated (*: *p* < 0.05, **: *p* < 0.01, ***: *p* < 0.001) after application of two-way ANOVA followed by Tukey.

To compare the expression levels of the viral proteins, immunoblot analysis was performed using anti-rabies G and N antibodies (Fig. 2a and Supplemental Fig. S1), and for comparison, values were normalized to those of the housekeeping protein, glyceraldehyde-3-phosphate dehydrogenase (GAPDH) (Fig. 2b). In HEP-infected cells, G and N proteins were detected from 1 d.p.i., but afterwards the amounts subsequently did not change appreciably up to 4 d.p.i. On the other hand, in HEP-10V- and HEP-30V-infected cells, expression of the G and N proteins appeared to increase from 2 to 4 d.p.i. The expression of those proteins in HEP-10V- and HEP-30V-infected cells were significantly increased from 2 to 4 d.p.i. compared in HEP-infected cells (*p* < 0.001). Notably, the N protein accumulated to significantly higher levels in HEP-30V-infected cells than in HEP-10V-infected cells at 2 and 3 d.p.i. (*p* = 0.02 and *p* < 0.001).

### Comparison of antigenicity between HEP and Vero-adapted HEPs

**Figure 3:**
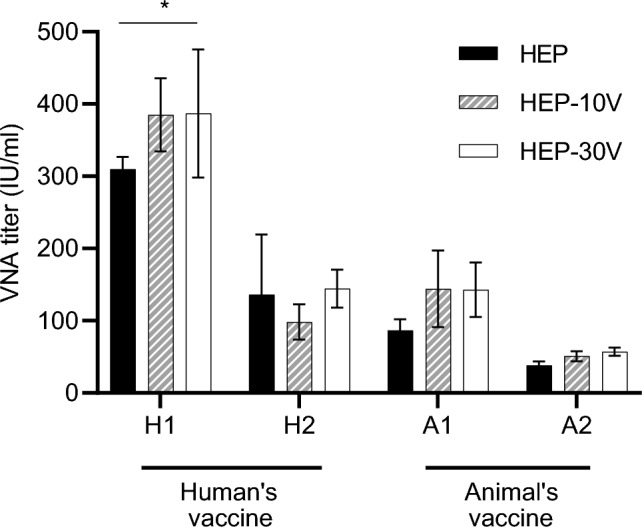
Comparison of virus-neutralizing antibody titers against HEP, HEP-10V and HEP-30V using vaccinated rabbit sera. Sera were collected from each of four rabbits (H1, H2, A1, A2). H1 and H2 were inoculated with the human-RABV vaccine (Rabipur, GSK Biologicals, Wavre, Belgium) and A1 and A2 were inoculated with the animal-RABV vaccine (KMB, KM Biologics, Kumamoto, Japan). Virus-neutralizing antibody (VNA) titers were determined by rapid fluorescent focus inhibition tests (RFFITs) and quantified using the World Health Organization (WHO) international units (IU/ml). Means and S.D. were calculated from four independent experiments. Significant difference is indicated (*: *p* < 0.05) in the neutralizing activities against HEP and HEP-30V in the H1 rabbit serum after application of two-way ANOVA followed by Tukey.

To compare the antigenicity of the parent and adapted viruses, rapid fluorescent focus inhibition tests (RFFITs) were performed using sera obtained from four rabbits (H1, H2, A1, and A2) (Fig. 3). The H1 and H2 sera were collected from rabbits vaccinated with a human rabies vaccine (Rabipur) and the A1 and A2 sera were collected from rabbits vaccinated with an animal rabies vaccine (KMB, KM Biologics, Kumamoto, Japan). Although a statistically significant difference in VNA titers was observed between HEP and HEP-30V (*p* = 0.048) using one serum (H1), statistically significant differences were not detected for any comparisons using other tested sera.

### Comparison of pathogenicity between HEP and adapted HEPs

**Figure 4:**
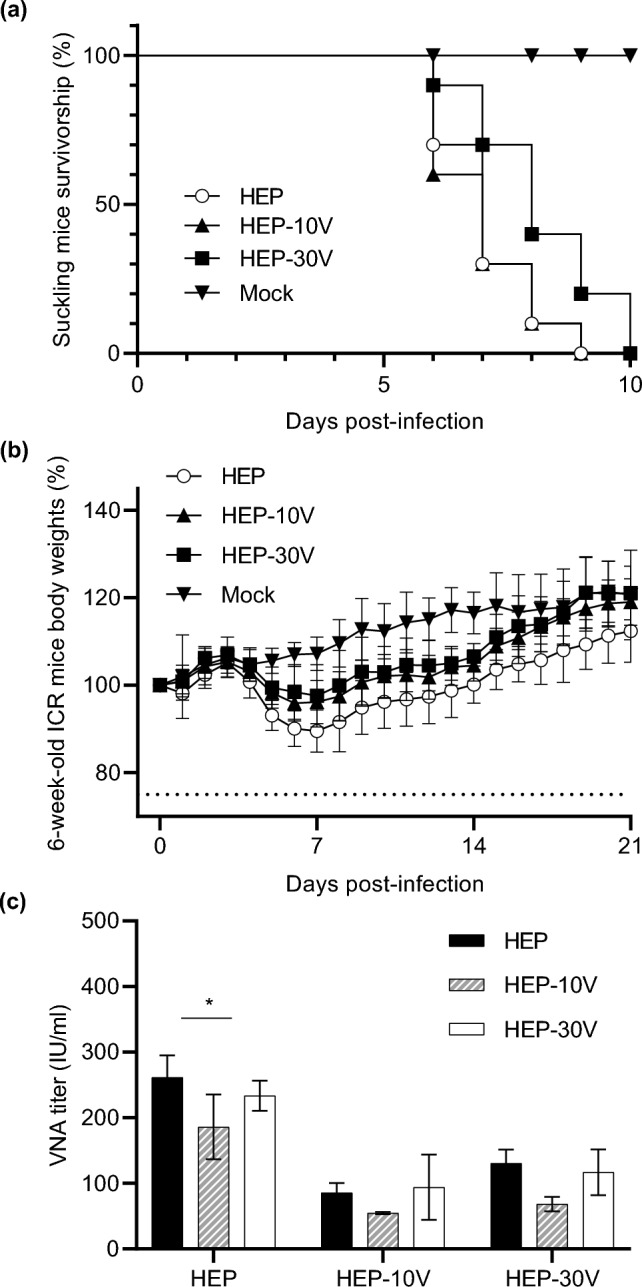
Comparison of pathogenicity among HEP, HEP-10V and HEP-30V in suckling and 6-week-old mice, and VNA titers against HEP-Flury using sera pooled from each infected-adult mice group. Suckling (n = 10/group) and 6-week-old mice (n = 8/group) were inoculated by intracerebral injection with 10^5^ FFU per mouse of the respective virus, or with an equivalent volume of medium (mock). Plots show the survival rate of suckling mice using a Kaplan–Meier plot (**a**) and relative body weights (normalized to baseline) of 6-week-old mice (**b**). Body weight data are presented as mean and error bars represent the S.D. of each group. (**c**) The mouse sera were collected from surviving 6-week-old mice which were infected by intracerebral route with HEP, HEP-10V or HEP-30V. VNA titers were determined by RFFIT and VNA titers are quantified using IU/ml. Means and S.D. was calculated from three independent experiments. Error bars represent the S.D. of each group. Significant difference is indicated (*: *p* < 0.05) in the neutralizing activity between HEP vs HEP-10V on the inoculated HEP mice group after application of two-way ANOVA followed by Tukey.

To compare the pathogenicity of HEP and the adapted strains, 10^5^ focus-forming unit (FFU) of HEP, HEP-10V, and HEP-30V were inoculated intracerebrally into suckling ICR mice (Fig. 4a) and 6-week-old adult ICR mice (Fig. 4b). Suckling mice infected with HEP or HEP-10V began to show neurological signs (such as tremors and paralysis) at 4 d.p.i., subsequently dying at 6–9 d.p.i. However, HEP-30V-infected suckling mice presented neurologic signs at 5 d.p.i., subsequently dying at 6–10 d.p.i. For all strains, all infected adult mice survived until 30 d.p.i., and showed no clinical signs other than body weight loss. Among the inoculated adult mice, body weights monitored through 21 d.p.i., in those infected with HEP exhibited a mean loss of weight (compared to baseline) of 10.6% at 7 d.p.i. before subsequently recovering. In contrast, HEP-10V- or HEP-30V-infected mice exhibited mean weight losses of 4.0% or 2.6%, respectively, at 7 d.p.i. before subsequently recovering.

### Comparison of VNA activity using sera from mice infected with HEP or adapted HEPs

Serum was collected from mice inoculated with each of the viral strains. Since the amounts of serum from individual mice were not sufficient for the tests, equal volume of sera from each mouse from a given group were pooled for analysis by RFFIT (Fig. 4c). Sera from mice infected with HEP showed that nominally highest VNA titers against HEP, with the differences achieving statistical significance against HEP-10V (*p* = 0.020). In sera from HEP-10V- and HEP-30V-infected mice, there was no significant difference in VNA titers against HEP, HEP-10V, or HEP-30V.

### Comparison of the viral genomes among HEP, HEP-10V, and HEP-30V

**Figure 5:**
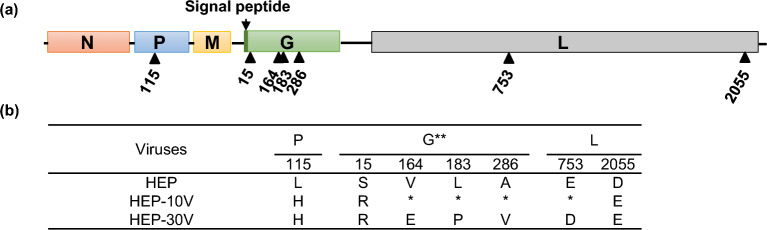
Comparison of amino acid sequences between HEP, Vero-adapted viruses. (**a**) Schematic of the rabies genome and rectangles indicate the open reading frames for the indicated proteins. Change of amino acids and their positions are shown by arrowheads. (**b**) Sequence details of the adapted viruses that are described in the present study. Amino acids are indicated by the standard one-letter abbreviations. *: Amino acid sequence identical with HEP. **: Amino acid positions are numbered based on the mature G protein without signal peptide.

Sequence analysis revealed that the nucleotide sequences of HEP-10V (Accession No. LC785440) and HEP-30V (Accession No. LC785441) exhibited 99.97% and 99.94% identity to those of HEP (Accession No. LC785439). All of the observed changes were missense; no silent mutations were detected. The positions of AA substitutions in the viral genome are indicated in Fig. 5a, and included totals of 3 and 7 AA substitutions for the proteomes of HEP-10V and HEP-30V, respectively (Fig. 5b). In HEP-10V, one AA substitution each was found in the P, G, and L proteins. In HEP-30V, a further three AA substitutions were found in the G protein, as well as one AA substitution in the L protein.

### Comparison of viral growth in Vero cells of recombinant HEP and HEP-10V viruses

**Figure 6:**
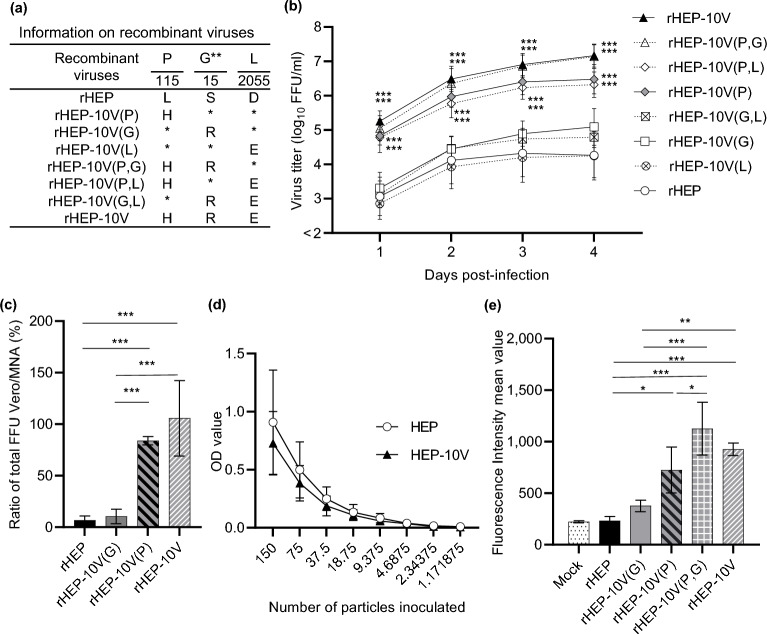
Efficiency of viral growth, entry, and expression of G protein of each recombinant viruses in Vero cells. (**a**) Information on recombinant viruses used in this figure. *: Amino acid sequence identical with rHEP. **: Amino acid positions are numbered based on the mature G protein without signal peptide. (**b**) Vero cells were inoculated with the indicated recombinant viruses (**a**) at a M.O.I. of 0.05 and supernatants were collected every day until 4 d.p.i. Viral titers were determined in MNA cells. (**c**) The total number of focuses was determined by counting the number of focuses stained with the FITC anti-rabies monoclonal globulin in Vero or MNA cells under a fluorescence microscope. The ratio of the number of focuses in Vero to that in MNA was compared. (**d**) The particle titer of each secreted alkaline phosphatase (SEAP)-expressing VSVp stock was determined in MNA cells. Vero cells then were inoculated with two-fold serial dilutions (starting from 150 particles) of VSVp pseudotyped with the HEP or HEP-10V G protein SEAP activity was assessed in culture supernatants and detected by optical density (OD). (**e**) Vero cells were inoculated with each recombinant virus at an M.O.I. of 5 and harvested at 2 d.p.i. The cells were stained with anti-rabies G protein monoclonal antibody (#7-1-9) and FITC-conjugated anti-mouse secondary. After that, cells were fixed with 4% paraformaldehyde, and finally analyzed by BD FACS Canto II flow cytometer (Becton Dickinson and Company; BD, Franklin Lakes, NJ, USA) and Kaluza analysis software Version 2.1 (Beckman Coulter Life Sciences, Indianapolis, IN, USA). Data are presented as the mean and S.D. from three (**b**, **d**, **e**) or four (**c**) independent experiments. Significant differences are indicated in the comparison between rHEP and each recombinant virus after application of two-way ANOVA followed by Tukey (**b**), or each virus after application of one-way ANOVA followed by Turkey (**c**, **e**) or two-way ANOVA followed by Sidaks (**d**) (*: *p* < 0.05, **: *p* < 0.01, ***: *p* < 0.001).

To determine which substitutions are responsible for the adaptation of viral growth in Vero cells, we employed reverse genetics to construct eight recombinant viruses corresponding to HEP, HEP-10V and HEP with each of one or two mutations (Fig. 6a). The resulting recombinant viruses were inoculated into Vero cells at an M.O.I. of 0.05, and viral growth was compared by measuring viral titers at multiple time points (Fig. 6b and Supplemental Table S3). Among the tested strains, the recombinant HEP (rHEP) strain showed the lowest titers during propagation in Vero cells. rHEP-10V(L), which had L(D2055E) in the L protein, yielded titers that were statistically indistinguishable from those of the parent rHEP. rHEP-10V(G), which had G(S15R) in the mature G protein, and the rHEP-10V(G,L), which had both G(S15R) and L(D2055E), yielded titers that were nominally higher than those obtained with rHEP, although the differences were not statistically significant. On the other hand, rHEP-10V(P), which had P(L115H) in the P protein, and rHEP-10V(P,L), which had both P(L115H) and L(D2055E), yielded titers that were significantly higher than those of rHEP (*p* < 0.001) at all assessed time points. The rHEP-10V(P,G), which had both P(L115H) and G(S15R), yielded titers that were nominally higher than those of rHEP-10V(P) and rHEP-10V(P,L). Titers obtained from rHEP-10V(P,G) were statistically indistinguishable from those obtained from rHEP-10V. Interestingly, cultures of both rHEP-10V(P,G) and rHEP-10V showed CPE at 3 or 4 d.p.i. in Vero cells, an observation not seen with the other recombinant viruses (data not shown).

### Comparison of virus entry in Vero cells by recombinant viruses

To compare the efficiency of entry by recombinant viruses, rHEP-10V(P) with P(L115H) and rHEP-10V(G) with G(S15R) were used to infect both Vero and MNA cells and following adsorption, the infected cells were overlaid with methylcellulose medium. Following staining with FITC-conjugated anti-rabies virus antibody, the cultures were analyzed for the number of foci, and the ratio of foci in the two cell lines was calculated (Fig. 6c). This analysis demonstrated that the entry of rHEP-10V(P) and rHEP-10V into Vero cells were statistically significantly more efficient than that of rHEP and rHEP-10V(G) (*p* < 0.001). On the other hand, the entry of rHEP-10V(G) into Vero cells was statistically indistinguishable from that of rHEP.

To examine the role of the G(S15R) substitution, viral entry was assessed using secreted alkaline phosphatase (SEAP)-expressing pesudotyped vesicular stomatitis virus (VSVp) pesudotyped with the G protein of either HEP or HEP-10V. Before initiation of this experiment, we tittered the number of particles of the VSVp stock by inoculating MNA cells and detecting particles by indirect fluorescence assay using anti-VSV N antibody. Based on these titers, Vero cells were inoculated with a series two-fold dilution of each VSVp starting from 150 particles per well. The results showed that similar rates of Vero cell entry were observed with recombinant VSVp pseudotyped with the parental HEP or the adapted HEP-10V G protein (Fig. 6d).

### Comparison of cell surface accumulation of viral proteins by recombinant viruses

The results described above showed that rHEP-10V(P) entered Vero cells more efficiently than rHEP, which could not be attributed to differences in the G protein sequence. We hypothesized that the observed difference in entry efficiency might be attributable to the amount of G protein on the viral particles when comparing among recombinant viruses. To assess the level of G protein on the surfaces of Vero cells, the recombinant viruses rHEP, rHEP-10V(G), rHEP-10V(P), rHEP-10V(P,G), and rHEP-10V, were inoculated to Vero cells at an M.O.I. of 5. At 2 d.p.i., the infected cells were collected and stained with anti-rabies G protein monoclonal antibody (#7-1-9). Analysis by fluorescence-activated cell sorting (FACS) showed that the level of G protein displayed on the cell surface was highest in rHEP-10V(P,G)-infected cells, followed by those in rHEP-10V-, rHEP-10V(P)-, rHEP-10V(G)-, and rHEP-infected cells (Fig. 6e). In the rHEP-infected cells, the amount of G protein displayed was lower as well as that in mock-infected cells (*p* = 0.815), while that on rHEP-10V(P)-infected cells exceeded those on rHEP-10V(G)- (*p* = 0.095) and rHEP-infected cells (*p* = 0.012). Interestingly, the level of G protein on rHEP-10V(P,G)-infected cells was significantly greater more than that in rHEP-10V(P)-infected cells (*p* = 0.044).

### Comparison of viral growth among recombinant HEP-10V with each mutation in HEP-30V

**Figure 7:**
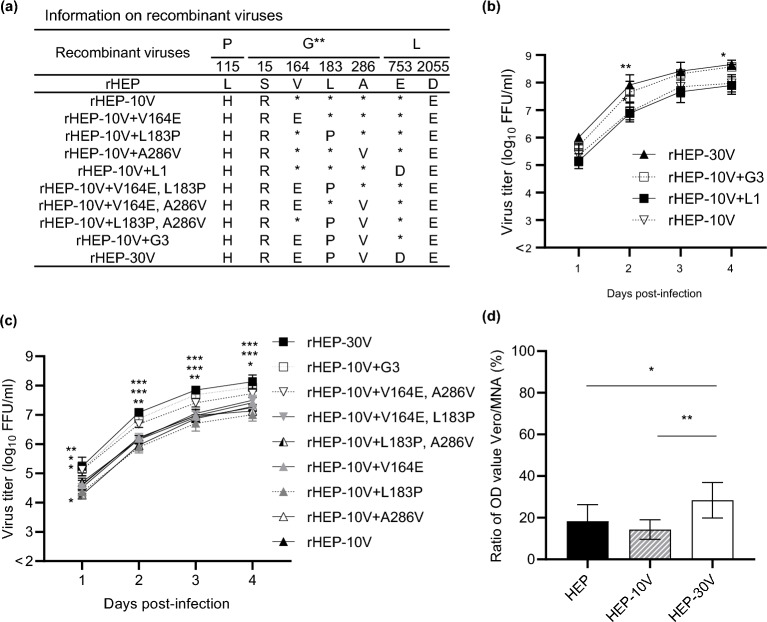
Comparison of viral growth in Vero cells among recombinant HEP strains, and entry of VSVp pseudotyped with the G protein of HEP, HEP-10V or HEP-30V. (**a**) Information on recombinant viruses used in this figure. *: Amino acid sequence identical with rHEP. **: Amino acid positions are numbered based on the mature G protein without signal peptide. Four recombinant viruses were infected to Vero cells at an M.O.I. of 0.05 (**b**), while nine recombinant viruses were inoculated at an M.O.I. of 0.01 (**c**). Viral titers were determined in MNA cells. The means and S.D. of the log_10_ of the viral titers are calculated from three independent experiments. Significant differences are indicated in the comparison between rHEP-10V and recombinant viruses after application of two-way ANOVA followed by Tukey (*: *p* < 0.05, **: *p* < 0.01, ***: *p* < 0.001). (**d**) Vero and MNA cells then were inoculated with 150 particles of VSVp pseudotyped with the G protein of HEP, HEP-10V, or HEP-30V. Following growth, SEAP activity was assessed in culture supernatants and detected by OD value, and the ratio of values in Vero and MNA cells was calculated. Means and S.D. were calculated from four independent experiments. Significant differences are indicated (*: *p* < 0.05, **:* p* < 0.01) after application of one-way ANOVA followed by Tukey.

Compared to HEP-10V, HEP-30V harbors 4 additional mutations, resulting in three AA substitutions in the G protein, G(V164E), G(L183P), and G(A286V), and one AA substitution in the L protein, L(E753D). To investigate the effect of these substitutions on viral growth, we generated recombinant viruses using reverse genetics to introduce these HEP-30V mutations into the genome of HEP-10V (Fig. 7a). In an initial experiment, we compared the viral growth of rHEP-10V, rHEP-10V harboring the single mutation in the L protein (rHEP-10V+L1), rHEP-10V harboring the triad of mutations in the G protein (rHEP-10V+G3), and rHEP-30V (Fig. 7b and Supplemental Table S4). The results indicated that the change in the L protein did not affect viral growth, while the three mutations in the G protein, whether in rHEP-10V+G3 or in rHEP-30V, provided significantly enhanced viral growth compared to the rHEP-10V parent (rHEP-10V+G3; *p* = 0.028, rHEP-30V; *p* = 0.001, 0.039, respectively).

Next, recombinant viruses harboring individual or paired mutations in the G protein were constructed (Fig. 7a) and their viral growth was compared to that of the rHEP-10V parent (Fig. 7c and Supplemental Table S5). Among the resulting mutants, only the V164E, A286V double mutant (rHEP-10V+V164E, A286V) exhibited significantly more efficient growth than rHEP-10V (*p* = 0.049, 0.003, 0.010, and 0.014, respectively), and the other recombinant viruses with single or double substitutions grew as well as rHEP-10V. However, the viral growth of rHEP10V+V164E, A286V was significantly attenuated compared to those of rHEP-30V at 3 d.p.i. (*p* = 0.036).

### Comparison of viral entry using VSVp pseudotyped with the G protein of HEP-30V

Recombinant viruses rHEP-10V+G3 and rHEP-30V, both of which harbored three mutations in the G proteins exhibited significantly improved growth compared to rHEP-10V (Fig. 7c). As above, we compared viral entry efficiencies using SEAP-expressing VSVp pesudotyped with the G protein of either HEP-10V or HEP-30V. The results indicated that the VSVp pseudotyped with the HEP-30V G protein entered Vero cells with significantly greater efficiency than did VSVp pseudatyoed with HEP G protein (*p* = 0.029) or with the HEP-10V G protein (*p* = 0.006) (Fig. 7d).

### Comparison of viral growth among recombinant viruses with five mutations

**Figure 8:**
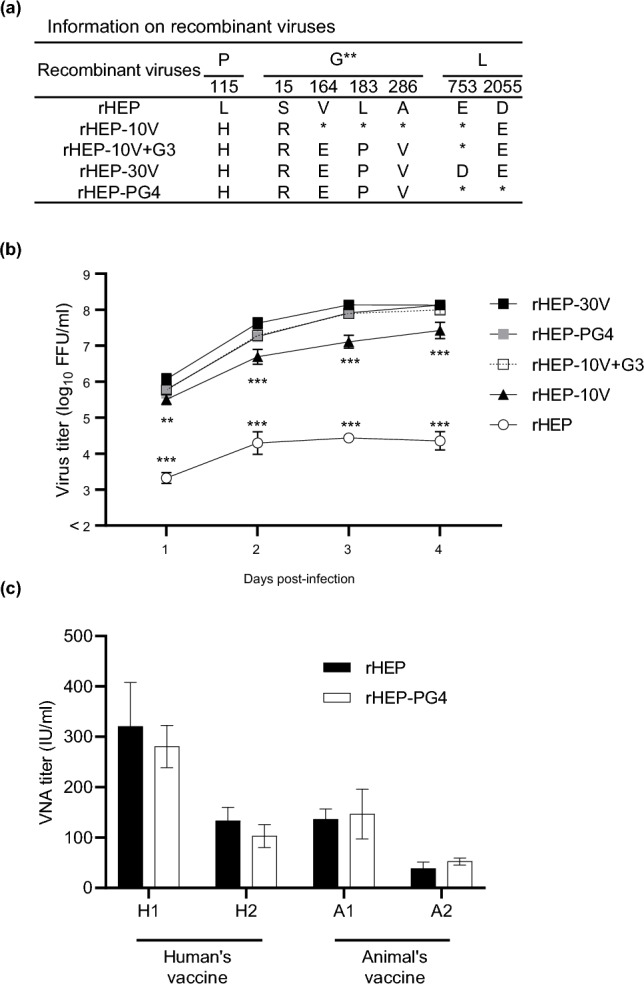
Viral growth in Vero cells of recombinant HEP with 5 amino acid substitutions identified in Vero-adapted strains, named rHEP-PG4. (**a**) Information on recombinant viruses used in this figure. *: Amino acid sequence identical with rHEP. **: Amino acid positions are numbered based on the mature G protein without signal peptide. (**b**) Vero cells were inoculated with the recombinant strains at an M.O.I. of 0.05. Viral titers were determined in MNA cells. The means and S.D. of the log_10_ of the viral titer calculated from three independent experiments. Significant differences are indicated in the comparison between rHEP-30V and each strain after application of two-way ANOVA followed by Tukey (**: *p* < 0.01, ***: *p* < 0.001). rHEP-30Vvs rHEP-10V+G3, rHEP-PG4 had no significant difference at all time points after application of two-way ANOVA followed by Tukey (*p* > 0.05). (**c**) Virus-neutralizing antibody (VNA) titers were determined by RFFITs using rabbit serum and quantified using IU/mL. Means and S.D. were calculated from three independent experiments. No significant difference is indicated (*p* > 0.05) in the neutralizing activities against rHEP and rHEP-PG4 after application of two-way ANOVA followed by Tukey.

In the experiments described above, we determined that mutations resulting in five substitutions (P(L115H), G(S15R), G(V164E), G(L183P) and G(A286V)) all contributed to the potentiation of growth efficiency in Vero cells. To confirm these results, we constructed a recombinant virus, designated rHEP-PG4, that carries all five of these mutations (Fig. 8a) and assessed the viral growth of this strain in Vero cells (Fig. 8b and Supplemental Table S6). We observed that the growth of rHEP-PG4 was statistically indistinguishable from those of rHEP-10V+G3 and rHEP-30V. This result showed that this rHEP-PG4 strain represents a version of HEP-Flury with the minimum number of mutations sufficient for Vero cell adaptation. To compare the antigenicity of the rHEP and rHEP-PG4, RFFITs were performed using four sera from rabbits inoculated with rabies vaccines (H1, H2, A1, and A2) (Fig. 8c). There was no statistically significant differences in VNA titers against rHEP and rHEP-PG4.

## Discussion

In the present study, we succeeded in producing two Vero cell-adapted HEP-Flury strains, HEP-10V and HEP-30V. These strains had similar or lower pathogenicities than the parent HEP (Fig. 4a, b), and their antigenicities were similar to that of the parent (Figs. 3 and 4c). Nucleotide sequence analysis showed that the HEP-10V harbored mutations resulting in 3 AA substitutions, including P(L115H), G(S15R), and L(D2055E). HEP-30V carried additional mutations, which resulted in another three substitutions in the G protein, G(V164E), G(L183P) and G(A286V), and a single substitution in the L protein, L(E753D) (Fig. 5). This HEP-30V harboring a total of seven substitutions (compared to the parent, HEP) should be considered a candidate for use as a seed for vaccine production in Vero cells, given its adaptation to growth in Vero cells while exhibiting pathogenicity and antigenicity similar to those of the HEP parent.

Next, we examined the mechanism of adaptation by analysis of recombinant HEP carrying individual mutations. The recombinant viruses rHEP-10V(P), rHEP-10V(P,L), rHEP-10V(P,G), and rHEP-10V, of which carried a mutation in the P protein resulting in P(L115H) substitution, showed significantly better growth in Vero cells than did the strains lacking this mutation (rHEP, rHEP-10V(L), rHEP-10V(G) and rHEP-10V(G,L), respectively) (Fig. 6b and Supplemental Table S3). These results indicated that the P(L115H) substitution supports adaptation of HEP to growth in Vero cells. In addition, the rHEP-10V(P) demonstrated significantly more efficient entry into Vero cells than rHEP (Fig. 6c), indicating that the P(L115H) substitution potentiated effective viral entry. Furthermore, the amount of the G protein on the cell surface was increased in cells infected by virus harboring the mutation causing the P(L115H) substitution (Fig. 6e). Taken together, these results suggest that the P(L115H) substitution may increase the efficiency of cell entry by promoting the accumulation of the G protein on the cell surface and presumably on the surface of the resulting virus particle.

P protein has at least six major functions^9^ and one of which interacted with focal adhesion kinase (FAK) to regulate RABV infection^9,36^. FAK is a cytoplasmic tyrosine kinase that localizes to cellular focal contacts and plays important roles in cellular signaling pathways that are involved in the regulation of transcription, the progression of the cell cycle, the modulation of apoptosis, the control of cell migration, and the metastasis of transformed cells^36–38^. During RABV infection, FAK enhances viral growth by interacting with the P protein, leading to enhancement in the replication and/or translation of the viral RNA^36^. Within the P protein, AA residues between 106 and 131 have been associated with FAK binding; residues R106, R109, R113, F114, W118, and I125 are known to be required for this interaction^36^. The present work indicated a role for P protein residue 115, although there are no reports of a possible function for this AA. We conjecture that this residue may participate in the interaction between FAK and the P protein, such that the P(L115H) substitution enhances the accumulation of G protein on the surface of RABV-infected Vero cells. Further experiments will be required to clarify the role of the, P(L115H) substitution in RABV adaptation to the Vero cell line.

To confirm the reproducibility of these adaptations, recombinant HEP was inoculated into Vero cells and passaged using the same method. The recombinant HEP strain also showed CPE at passage 3, and then the supernatant was inoculated Vero cells for repeated passaging. rHEP passaged 10 times also harbored a total of three missense mutations. While these changes corresponded to one substitution each in the P, G, and L proteins, the positions of the substitutions differed from HEP-10V. Specifically, this strain (rHEP passaged 10 times) substitutions consisted of P(V105D), G(D211N), and L(K875T). The P(V105D) substitution would lie close to the substitution at residue 115 that was observed in HEP-10V. We infer that a domain of the P protein spanning residues 105–115 is important for the adaptation of RABV to growth in Vero cells.

The recombinant viruses rHEP-10V(G), rHEP-10V(G,L), rHEP-10V(P,G), and rHEP-10V, of which carried a mutation in the G protein resulting in G(S15R) substitution, showed better growth in Vero cells than the strains lacking this mutation (rHEP, rHEP-10V(L), rHEP-10V(P) and rHEP-10V(P,L), respectively) (Fig. 6b and Supplemental Table S3). On the other hand, no significant difference in entry was detected between rHEP and rHEP-10V(G), or between VSVp particles pseudotyped with G proteins from HEP and HEP-10V (Fig. 6c, d). We also observed that G protein accumulated to higher levels on the surface of Vero cells infected with recombinant viruses encoding the G(S15R) mutant protein (rHEP-10V(G) and rHEP-10V(P,G)) than on those infected by viruses lacking this mutation (rHEP and rHEP-10V(P), respectively) (Fig. 6e). Together, these results indicated that the G(S15R) substitution plays important roles in the adaptation of viral growth to Vero cells and in the accumulation of G protein on the cell surface, but does not provide any change in viral entry. We hypothesize that the G(S15R) substitution enhances viral budding from the surface of Vero cells by increasing the amount of G protein accumulating on the cell surface. Further analysis will be needed to resolve the role of the G(S15R) substitution in RABV propagation in Vero cells.

Compared to HEP-10V, HEP-30V had three additional AA substitutions including G(V164E), G(L183P) and G(A286V). rHEP-10V with all three substitutions, rHEP-10V+G3, grew better in Vero cells than strains lacking these mutations (rHEP-10V and rHEP-10V+L1) (Fig. 7b and Supplemental Table S4). In addition, VSVp particles pseudotyped with the G protein of HEP-30V exhibited more-efficient entry into Vero cells than those of HEP and HEP-10V (Fig. 7d). Recombinant viruses with only one or two of substitutions in the G protein exhibited growth in Vero cells similar to that seen with rHEP-10V (Fig. 7c and Supplemental Table S5). Only rHEP-10V+V164E, A286V exhibited growth in Vero cells that was significantly better than that seen with rHEP-10V, while this strain did less than by rHEP-10V+G3 and rHEP-30V. These results indicated that all three substitutions play important roles in viral growth, and that these changes are mediated by effects on virus entry.

The mature G protein consists of three domains (ectodomain, transmembrane domain and cytoplasmic domain)^9^, and all of the G protein substitutions identified in HEP-30V in the present work are located in the ectodomain. G protein residues 164, 183, and 286 have not identified as part of a pathogenicity-related site^19,39–41^, epitope site^42,43^, *N*-glycosylation site^40,42,44,45^, or cell receptor-binding site^10,46^. Other work has shown that the G protein recognizes and adheres to neuronal cell receptors such as heparan sulfate^47^, the acetylcholine receptor (nAChR)^48^, the neural cell adhesion molecule (NCAM)^49^, the low-affinity neurotrophic receptor (p75NTR)^50^ and the metabotropic glutamine receptor subtype II (mGluR2)^51^. In the present study, the G(V164E), G(L183P), and G(A286V) substitutions were associated with enhanced entry into Vero cells. Notably, Vero cells are known to display two receptors, NCAM and heparan sulfate, on the cell surface^49,52^. To our knowledge, the site within the G protein that is responsible for receptor-binding to NCAM has not been reported yet. On the other hand, the G protein site responsible for binding to the heparan sulfate receptor has been reported to be located at residues 126–273^47^. Therefore, two of the identified here (G(V164E) and G(L183P)) may contribute to changes in heparan sulfate binding by the G protein. In addition, in silico structural modeling of the G protein using Molecular Operating Environment software version 2022.02 (Chemical Computing Group, Montreal, Canada)^53^ suggests that residues 164 and 183 are located at conformationally related positions on the protein, whereas residue 286 is predicted to be located internal to the protein (not shown). Therefore, the G(V164E) and G(L183P) substitutions are candidates for sites that might enhance the binding of the G protein to cell receptors displayed on Vero cells, including heparan sulfate and other unknown receptors.

In comparison with the other Vero-adapted RABV isolate, strain PV-2061, a single amino acid residue proline (P) at position of 183 of the G protein of PV-2061 was identical to G(L183P) of HEP-30V. However, this proline at position of 183 is conserved in the parental strain PV and the other street viruses. In HEP, this amino acid is important for Vero adaptation, but it may have a different role in the other strains. Interestingly, PV-2061 has a P(L115F) substitution in the P protein in comparison with the parent strain PV. Since the substitution P(L115H) in HEP plays an important role in viral growth in Vero cells, the P(L115F) substitution might enhance viral growth of PV-2061 in Vero cells. Further experiments will be required to clarify the role of the P(L115H) substitution in the adaptation of RABV to the Vero cell line.

Thus, in the present work, we generated recombinant HEP-Flury by reverse genetics and demonstrated that the combination of five AA substitutions (P(L115H), G(S15R), G(V164E), G(L183P) and G(A286V)) were sufficient for the adaptation of this RABV strain to growth in Vero cells. This recombinant RABV, which have designated rHEP-PG4, maintained antigenicity similar to original HEP. In conclusion, rHEP-PG4 is expected to serve as a candidate seed virus for propagation in Vero cells for vaccine production.

## Materials and methods

### Cells

Vero cells (JCRB9013; Japanese Cancer Research Resources Bank, Tokyo, Japan) were maintained at 37 °C in Dulbecco’s modified Eagle’s minimum essential medium (DMEM) (Thermo Fisher Scientific, Waltham, MA, USA) supplemented with 5% of heat-inactivated fetal bovine serum (FBS) (Gibco, Grand Island, NY, USA). MNA cells were grown at 37 °C in Eagle’s minimum essential medium (MEM) (Thermo Fisher Scientific) supplemented with 10% heat-inactivated FBS. Baby hamster kidney fibroblast (BHK-21) cells were kindly provided by Dr. K. Morimoto (Yasuda Women’s University, Hiroshima, Japan) and BHK-21 stably expressing T7 RNA polymerase (BHK/T7-9) cells were provided by Dr. N. Ito (Gifu University, Gifu, Japan)^54^. BHK-21 cells, BHK/T7-9 cells, and human embryonic kidney (HEK-293T) cells (CRL-3216; American Type Culture Collection (ATCC), Manassas, VA, USA) were maintained at 37 °C in MEM supplemented with 10% heat-inactivated FBS.

### Viruses

RABV used in this study is our laboratory strain of HEP-Flury (HEP)^55^, which historically has been propagated in the MNA cells. The HEP strain originally was stocked in the Department of Veterinary Science, National Institute of Infectious Diseases, after two rounds of propagation on MNA cells. The complete genomic sequence of our strain, determined as part of the current work, has been deposited in the DNA Data Bank of Japan (DDBJ) as Accession Number: LC785439 (Supplemental Table S1).

### Adaptation of HEP-Flury strain to propagation in the Vero cell line

HEP was adsorbed to Vero cells in a T25 flask (Sumitomo Bakelite, Tokyo, Japan) for 1 h at 37 °C in growth medium (DMEM supplemented with 5% FBS). After adsorption, the cells were incubated in the growth medium for 7 days at 37 °C. The passages of cells were repeated until CPE was observed. After the appearance of CPE, the supernatant was harvested and used as the inoculum for the next passage, as follows: the supernatant was inoculated to 80% confluent Vero cells, and incubated in maintenance medium (DMEM supplemented with 2% FBS) until CPE was again observed. For each such passage, a portion of the supernatant was stored at − 80 °C until further use. Virus obtained after 10 and 30 passages in Vero cells was designated HEP-Flury 10V (HEP-10V) and HEP-Flury 30V (HEP-30V), respectively.

### Virus titration

Viral titers were determined by a direct fluorescent test using MNA cells. MNA cells (4.0 × 10^4^ cells/well) in 96-well plates and incubated at 37 °C with 5% CO_2_ for 24 h. MNA cells in 96-well plates were inoculated with serial tenfold dilutions of virus and incubated at 37 °C for 2 days. Cells then were fixed with 80% acetone for 30 min and stained with FITC Anti-Rabies Monoclonal Globulin (FUJIREBIO, Tokyo, Japan). Antigen-positive foci were counted under a fluorescence microscope (Nikon, Tokyo, Japan) and quantified as FFU per milliliter.

### Comparison of viral growth

Vero cells in 6-well plates were inoculated with each of the RABV strains at a M.O.I. of 0.05 or 0.01. For the rHEP-10V+V164E, L183P strain’s titer was too low to perform the growth curves at an M.O.I. of 0.05. Therefore, Fig. 7c was performed at an M.O.I. of 0.01. After 1 h of adsorption, the cells were washed three times with DMEM and 2 mL of maintenance medium was added to each well. The supernatant was collected at the indicated time points. Each experiment was repeated independently two or three times.

### Observation of plaques

Vero cells in 6-well plates were infected with each virus or negative control (medium only) at a M.O.I. of 0.01. After 1 h of adsorption, the cells were washed three times with DMEM and 2 mL of maintenance medium was added to each well. At the indicated time points, the cells in a given were fixed with 80% acetone and stained with FITC Anti-Rabies Monoclonal Globulin and Evans blue (FUJIFILM Wako Pure Chemical Corporation, Osaka, Japan). The stained cells were visualized under a fluorescence microscope and quantified using NIS-Elements D version 5.20.00 imaging software (Nikon).

### Inoculation of rabbits with rabies vaccines

The experimental protocol was approved by the Committee for Animal Experimentation of the National Institute of Infectious Diseases (NIID) (Approval number 120083). All methods were carried out in accordance with relevant guidelines and regulations. All possible efforts were made to minimize the suffering of laboratory animals. During this portion of the animal studies, the rabbits were housed in the animal facility of the NIID. All methods are reported in accordance with ARRIVE guidelines.

Four young adult female Japanese white rabbits (body weight, 2–3 kg) (Kitayama Labes, Nagano, Japan) were inoculated intradermally with commercial rabies vaccines, consisting of either the “KMB” rabies vaccine (KM Biologics, Kumamoto, Japan) intended for use in animal or the “Rabipur” rabies vaccine intended for use in human. Inoculation was performed every other week for a total of 7 doses, and blood samples were collected 7 days after the last immunization. Rabbits in this study were sacrificed via exsanguination under deep anesthesia by intravenous injection of Pentobarbital.

### Challenge experiment in mice with each HEP

The experimental protocol was approved by the Committee for Animal Experimentation of the National Institute of Infectious Diseases (NIID) (Approval number 121021). All methods were carried out in accordance with relevant guidelines and regulations. All possible efforts were made to minimize the suffering of laboratory animals. During this portion of the animal studies, all mice were housed in the animal facility of the NIID. All methods are reported in accordance with ARRIVE guidelines.

Six-week-old ICR (adult) mice (8/group) or suckling mice (10/group) (Japan SLC, Shizuoka, Japan) were inoculated intracerebrally with 10^5^ FFU/mouse of HEP, HEP-10V, HEP-30V, or MEM medium (as a negative control “mock”). Body weights of the adult mice were monitored until 21 d.p.i., and mortality was recorded once daily through for 30 d.p.i. adult mice or through 10 d.p.i. suckling mice. At 30 d.p.i., sera were collected from surviving adult mice. Mice in this study were sacrificed via isoflurane inhalation overdose euthanasia systems, followed by cervical dislocation.

### Reverse transcription-polymerase chain reaction (RT-PCR)

RABV RNA was extracted from the supernatant of infected cells using the QIAamp Viral RNA Mini Kit (QIAGEN, Hilden, Germany) according to the manufacturer’s protocol. To generate cDNA, RT was performed as follows: 10 µL of template RNA and 1μL of Random-Primers (Promega, Madison, WI, USA) were combined, and the mixture was heated at 95 °C for 1 min. Subsequently, AMV Reverse Transcriptase (Promega), Recombinant RNasin Ribonuclease Inhibitor (Promega), and dNTP Mixture (TaKaRa Bio, Shiga, Japan) were added, and the mixture then was incubated at 42 °C for 45 min before being incubated at 95 °C for 5 min. The resulting reverse transcribed cDNA was used for the subsequent PCR amplification of fragments of the RABV genomes.

The cDNA templates were subjected to PCR using Platinum Taq DNA Polymerase High Fidelity (Thermo Fisher Scientific) and the respective primer sets. Detailed information on the PCR conditions and primer sequences are provided in Supplemental Table S7. Amplified products were subjected to gel electrophoresis on 1% agarose to confirm fragment sizes. The PCR products then were purified using the QIAquick PCR Purification Kit (QIAGEN) and used as templates for DNA sequence analysis.

### Sequence analysis

Purified PCR products corresponding to segments of the RABV genome were analyzed by Sanger sequencing method using the BigDye Terminator v3.1 Cycle Sequencing Kit (Thermo Fisher Scientific) followed by separation on an Applied Biosystems 3130xl machine (Thermo Fisher Scientific). Sequence assembly and further analysis was conducted using GENETYX Ver.15 software (GENETYX, Tokyo, Japan).

### Rapid fluorescent focus inhibition test (RFFIT)

The VNA titers were determined using a modified RFFIT assay^56–58^. Briefly, sera were diluted in a 96-well plate five-fold with MEM containing 2% FBS and an equal volume of virus suspension containing 50 of a 50% focus forming dose (50 FFD_50_) of RABV then was added to each well. After incubation at 37 °C for 90 min, 100 µL of the mixture was transferred into MNA cells (4.0 × 10^4^ cells/well) in 96-well plate and incubated at 37 °C with 5% CO_2_ for 24 h. The controls for this experiment included the WHO reference and negative sera. After 24 h, the cells were fixed with 80% acetone and stained with FITC Anti-Rabies Monoclonal Globulin.

### Immunoblot analysis

Vero cells in 6-well plates were infected with RABV or negative control (medium) at an M.O.I. of 5. Aliquots of infected cells were harvested each day and lysed with 100 µL/well of lysis buffer (0.1% sodium dodecyl sulfate, 1% sodium deoxycholate 1% Triton X-100, 10 mM Tris–HCl (pH 7.4), 150 mM sodium chloride, 0.5 mM ethylene diamine tetra acetic acid (EDTA)) on ice for 1 h. The extracts were centrifuged at 15,000 rpm for 30 min at 4 °C, and the resulting supernatants were stored at − 80 °C until use. The concentration of total protein in each supernatant was determined using TaKaRa BCA Protein Assay Kit (TaKaRa Bio) according to manufacturer’s instructions. Equal weights of total protein were separated on a NuPAGE 4–12% Bis–Tris Gel (Thermo Fisher Scientific) and transferred to an Immobilon-P Transfer membrane (Thermo Fisher Scientific). After 1 h of blocking with Blocking One (Nacalai Tesque, Kyoto, Japan), the membrane was reacted at room temperature for 1 h with primary antibodies consisting of anti-RABV-G (1:200), anti-RABV-N (1:1000), or GAPDH antibody (1:2000) (5G4; Hy Test, Danvers, Turku, Finland). Anti-RABV-G and -N rabbit sera were prepared as described previously^44^. The membranes were then washed with Tris-buffered saline-0.05% Tween 20 (BIO-RAD, Hercules, CA, USA) and incubated for 1 h at room temperature with the following horseradish peroxidase (HRP)-conjugated secondary antibodies: Goat anti-Rabbit IgG (H+L) Secondary Antibody (1:2000) (65-6120; Thermo Fisher Scientific) or Goat Anti-Mouse IgG1 (1:1000) (A90-205P; Abcam, Cambridge, UK). Finally, the membranes were stained with Peroxidase Stain DAB kit (Nacalai Tesque) or Chemi-Lumi One Ultra (Nacalai Tesque). Band intensity was quantified using ImageJ software (National Institutes of Health, Bethesda, MD, USA).

### Construction of plasmids for reverse genetics

To construct the infectious clone of RABV, PCR was performed using Prime STAR GXL (TaKaRa Bio) to generate three overlapping PCR amplicons covering the entire RABV genome. Overlapping PCR then was performed with three PCR amplicons using Phusion Hot Start II High-Fidelity DNA Polymerase (Thermo Fisher Scientific) to generate a cDNA representing the full genome. Mutant viruses were constructed by introducing point mutations by PCR using Prime STAR Max (TaKaRa Bio) and synthetic primers with the indicated sequences. Detailed information on the PCR conditions and the primer sequences are provided in Supplemental Tables S8 and S10. The assembled cDNA containing the sequence of the hammerhead ribozyme sequence (HamRz), the full-length cDNA of RABV genome in the antigenomic orientation, and the hepatitis delta virus ribozyme sequence (HdvRz) was inserted between the *Kpn*I and *Pst*I sites of the pcDNA3.1 Zeo (+) plasmid (Thermo Fisher Scientific), in parallel to the previously reported constructs^19,35^. Before the insertion into the cloning vector, the Zero Blunt TOPO PCR Cloning Kit (Thermo Fisher Scientific) was used for sub-cloning.

To construct helper plasmids, the genes encoding the N, P, G, and L proteins were amplified from HEP-Flury using conventional PCR (Supplemental Table S9) and cloned between the *Kpn*I and *Pst*I sites of the pcDNA3.1 Zeo (+) plasmid. The resulting helper plasmids were designated pH-N, pH-P, pH-G, and pH-L, respectively. Nucleotide sequences of the assembled full-length cDNA clones and of the helper plasmids were confirmed by sequencing.

### Rescue of recombinant viruses

BHK-21 or BHK/T7-9 cells (3.0 × 10^5^ cells/well) were grown overnight in 6-well plates in MEM supplemented with 10% FBS. Cells were transfected with per well 1.2 µg of the full-length plasmid and 450 ng each of pH-N, pH-P, pH-G, and pH-L, and transfection was conducted using the TransIT-LT1 Transfection Reagent (Mirus Bio, Madison, WI, USA) according to the manufacturer’s protocol. After 20–24 h, the transfection medium was replaced with fresh MEM supplemented with 10% FBS. After 5 days, supernatants were collected and an aliquot of each was inoculated to MNA cells to confirm the presence of the virus as assessed by a fluorescent antibody (FA) test. Supernatants from virus-positive wells were propagated in MNA cells to produce virus stock. Nucleotide sequences of all rescued viruses were confirmed by sequencing.

### Comparison of efficiency of cell infection

The FFU of the recombinant viruses were determined by plating in MNA cells. The resulting values were used to generate serial two-fold dilutions of recombinant viruses starting from 1000 FFU/well, and these dilutions were used as inoculation to infect MNA or Vero cells in 12-well plates. After 1 h of adsorption, the cells were washed three times with MEM and medium containing 1% methyl cellulose was added at 1 mL/well, and the resulting cultures were incubated for 2 days. The cells then were fixed for 30 min with 10% formaldehyde containing 0.4% Triton X-100 (Merck, Darmstadt, Germany) and stained with FITC Anti-Rabies Monoclonal Globulin. Antigen-positive foci were counted under a fluorescence microscope. The ratio of the number of foci in Vero cells to that in MNA cells was calculated in the well which inoculated with 500 FFU/well.

### Flow cytometric analysis

Vero cells in 6-well plates were infected with viruses or negative control (medium “mock” infection) at an M.O.I. of 5 and incubated for 2 days. The infected cells were washed with phosphate-buffered saline (PBS) and released by treatment with 2.9 mM EDTA. The cells were collected, washed with sorting buffer, and reacted for 1 h on ice with the mouse monoclonal anti-RABV G antibody #7-1-9^44^ (0.4 mg/mL) (1:80). Cells were then washed twice and reacted for 1 h on ice with the FITC-conjugated Goat Anti-Mouse IgG1 secondary antibodies (1:1600) (FI-2000; Vector Laboratories, Newark, CA, USA). Next, the cells were washed twice and fixed at room temperature for 20 min in 4% paraformaldehyde. Finally, the cells were again washed twice and sorted using a BD FACS Canto II flow cytometer (Becton Dickinson and Company; BD, Franklin Lakes, NJ, USA) with blue lasers (488 nm) for the detection of FITC. The resulting data were analyzed using Kaluza Analysis Software Version 2.1 (Beckman Coulter Life Sciences, Indianapolis, IN, USA).

### Production of pseudotyped vesicular stomatitis virus (VSVp)

Secreted alkaline phosphatase (SEAP)-expressing VSVp particles that were pseudotyped with HEP, HEP-10V, or HEP-30V G proteins were produced as described previously^59^. Briefly, an expression plasmid encoding the RABV G protein was transfected into 80% confluent HEK293T cells using polyethylenimine (Thermo Fisher Scientific) according to the manufacturer’s instructions. At 2 d.p.i., the cultures were inoculated with VSVΔG-SEAP (a kind gift of Dr. Y. Matsuura, Osaka University, Japan) at an M.O.I. of 1. After 1 h of adsorption, the HEK293T cells were washed three times with MEM and MEM containing 2% FBS was added to each well. After 24 h, the culture supernatants were harvested and filtered through gamma-sterilized Millex-HV Syringe Filter Units, (0.45 µm pore size, polyvinylidene fluoride (PVDF) membrane, 33 mm diameter) (Thermo Fisher Scientific). These resulting virus suspensions were stored at -80 °C until use.

### Titration of VSVp

To determine the infectious titer of the VSVp stocks, MNA cells in 96-well plates were inoculated with serial two-fold dilution of the VSVp suspensions. After 1 h of adsorption, MNA cells were washed three times with MEM and MEM containing 2% FBS was added at 100 µL/well. After overnight incubation, cells were fixed with 80% acetone for 30 min and incubated for 1 h at 37 °C with mouse anti-VSV nucleoprotein antibody (1:1000) (MABF2348; Thermo Fisher Scientific) as the primary antibody. After three washes, cells were stained with FITC-conjugated goat anti-mouse IgG (H&L; 1:200) (ab6785; Abcam) as the secondary antibody.

### Quantification of infection with VSVp

Serial two-fold dilutions of VSVp stocks were inoculated to MNA or Vero cells and adsorbed for 1 h. The cells then were washed three times with MEM or DMEM, and MEM or DMEM supplemented with 2% FBS was added at 100 µL/well. After overnight incubation at 37 °C, an aliquot 40 µL of each culture supernatant was transferred to a new 96-well plate, to which substrate solution (SIGMAFAST p-Nitrophenyl Phosphate Tablets, Thermo Fisher Scientific) was added at 200 µL/well. After the plates were incubated for 2 h at 37 °C, the amount of SEAP was determined by measuring the optical density at a wavelength of 405 nm (OD405) using a spectrophotometer (iMarkTM Microplate Absorbance Reader, BIO-RAD).

### Statistical analysis

Unless otherwise indicated, data are presented as the mean ± standard deviation (S.D.). Statistical analyses were conducted using a one-way Analysis of Variance (ANOVA) with post hoc Tukey’s multiple-comparison tests or the two-way ANOVA followed by either Tukey’s, Dunnett’s or Sidak’s multiple-comparison tests. A log-rank test was used to analyze the Kaplan–Meier survival curves. Analyses were performed using in GraphPad Prism9 (GraphPad Software, San Diego, CA, USA). Values of *p* < 0.05 were considered statistically significant.

### Supplementary Information

Supplementary Information.

## Data Availability

Sequence data that support the findings of this study have been deposited in the DNA Data Bank of Japan with Accession Numbers LC785439, LC785440 and LC785441. All data generated or analyzed during this study are included in this published article and its Supplementary Information files.
