# Supplementary material for: Construction of Vero cell-adapted rabies vaccine strain by five amino acid substitutions in HEP-Flury strain
Source: Sci Rep. 2024 May 31;14:12559. doi: 10.1038/s41598-024-63337-9 (PMC11143356; doi:10.1038/s41598-024-63337-9)
Supplement: Supplementary file 1 — Supplementary Information. [file 41598_2024_63337_MOESM1_ESM.pdf]

Title: Construction of Vero cell-adapted rabies vaccine strain by five amino acid substitutions in HEP-Flury strain

Authors:

Michiko Harada[1,2], Aya Matsuu[2], Eun-Sil Park[2], Yusuke Inoue[1,2], Akihiko Uda[2], Yoshihiro Kaku[2], Akiko Okutani[2], Guillermo Posadas-Herrera[2], Keita Ishijima[2], Satoshi Inoue[2], Ken Maeda[1,2*]

Author affiliations:

[1]Joint Graduate School of Veterinary Medicine, Yamaguchi University, Yamaguchi, Yamaguchi, Japan.

[2]Department of Veterinary Science, National Institute of Infectious Diseases, Shinjuku-ku, Tokyo, Japan.

*Corresponding Author:

Ken Maeda, DVM, PhD

ORCID: 0000-0002-3488-5439

1-23-1 Toyama, Shinjuku-ku, Tokyo 162-8640, Japan

Tel: +81-3-4582-2750

Email: kmaeda@niid.go.jp

Supplementary Table S1. Comparison of amino acid sequences between our laboratory's HEP-Flury (HEP) strain and those previously reported for HEP-Flury strains

Strain	P					M			G (signal peptide)		G**									L						
	28	130	191	289	295	16	106	172	14	15	40	120	164	206	236	259	273	484	504	75	383	745	1319	1653	1848	2091
HEP (LC785439)	I	S	G	D	T	T	A	D	F	P	G	H	V	T	M	N	E	P	R	D	L	Q	R	V	R	R
AB085828	*	*	*	*	*	*	*	*	S	*	*	*	*	*	*	*	*	*	*	*	*	*	*	*	*	*
LC717409	*	T	E	*	*	*	*	N	*	*	*	*	*	*	*	*	V	T	*	*	*	*	*	*	*	*
GU565704	*	T	E	*	K	*	V	N	*	S	*	*	*	*	*	*	V	T	*	G	S	*	*	*	*	S
LC717410	*	T	E	*	*	*	V	N	*	*	*	*	*	I	*	*	V	T	I	*	S	*	*	*	*	*
LC717411	V	T	E	*	*	*	V	N	*	*	*	*	E	I	V	D	V	T	I	*	S	*	K	I	*	*
LC717412	V	T	E	N	*	I	V	N	*	*	R	V	E	I	V	D	V	T	I	*	S	R	K	*	K	*

*: Amino acid identical with our laboratory HEP.

**: Position of amino acids in the mature G protein without the signal peptide.

Supplemental Table S2. Comparison of virus titers between HEP-Flury (HEP) and Vero-adapted HEP-Flury strains

Days post infection	Virus titers (log_{10} FFU/ml) (Mean±S.D.)		
	HEP	HEP-10V	HEP-30V
1	3.15±0.99	3.91±0.29	4.48±0.42
2	4.47±0.83	5.76±0.11	6.54±0.45
3	4.87±0.93	6.25±0.30	7.14±0.41
4	4.82±0.99	6.75±0.19	7.79±0.07

Supplemental Table S3. Comparison of virus titers among recombinant HEP-Flury strains to evaluate the effect of mutations in HEP-10V

Days post infection	Virus titers (log_{10}FFU/ml) (Mean±S.D.)							
	rHEP	rHEP-10V(P)	rHEP-10V(G)	rHEP-10V(L)	rHEP-10V(P,G)	rHEP-10V(P,L)	rHEP-10V(G,L)	rHEP-10V
1	3.03±0.55	4.81±0.48	3.28±0.45	2.83±0.46	5.04±0.36	4.77±0.21	3.10±0.39	5.25±0.30
2	4.08±0.68	5.96±0.31	4.43±0.35	3.89±0.63	6.35±0.43	5.75±0.40	4.45±0.36	6.47±0.36
3	4.28±0.67	6.39±0.37	4.88±0.36	4.16±0.72	6.85±0.32	6.23±0.34	4.73±0.31	6.90±0.32
4	4.23±0.64	6.47±0.42	5.07±0.53	4.21±0.70	7.13±0.34	6.31±0.36	4.78±0.29	7.16±0.33

Supplemental Table S4. Comparison of virus titers of the rHEP-10V to evaluate the effect of mutations on the G or L proteins in rHEP-30V

Days post infection	Virus titers (log_{10}FFU/ml) (Mean±S.D.)			
	rHEP-10V	rHEP-10V+G3	rHEP-10V+L1	rHEP-30V
1	5.35±0.37	5.69±0.10	5.13±0.26	6.00±0.09
2	6.92±0.36	7.64±0.39	6.87±0.23	7.90±0.37
3	7.83±0.57	8.31±0.06	7.66±0.18	8.40±0.32
4	7.96±0.31	8.57±0.09	7.88±0.31	8.65±0.16

Supplemental Table S5. Comparison of virus titers among rHEP-10 with the substitutions in rHEV-30V

Days post infection	Virus titers (log_{10}FFU/ml) (Mean±S.D.)								
	rHEP-10V	rHEP-10V+V164E	rHEP-10V+L183P	rHEP-10V+A286V	rHEP-10V+V164E, L183P	rHEP-10V+V164E, A286V	rHEP-10V+L183P, A286V	rHEP-10V+G3	rHEP-30V
1	4.69±0.13	4.61±0.05	4.35±0.20	4.58±0.17	4.5±0.14	5.12±0.10	4.26±0.03	5.14±0.25	5.23±0.31
2	6.14±0.14	6.21±0.15	5.89±0.19	6.21±0.08	6.16±0.04	6.68±0.11	5.96±0.15	6.86±0.08	7.08±0.08
3	6.91±0.09	6.96±0.14	6.70±0.27	6.97±0.20	7.04±0.19	7.40±0.14	6.86±0.05	7.68±0.22	7.84±0.05
4	7.24±0.10	7.41±0.20	7.00±0.21	7.13±0.14	7.49±0.17	7.72±0.13	7.28±0.06	7.93±0.11	8.13±0.23

Supplemental Table S6. Comparison of virus titers of the rHEP-PG4 with only five substitutions

Days post infection	Virus titers (log_{10}FFU/ml) (Mean±S.D.)				
	rHEP	rHEP-10V	rHEP-10V+G3	rHEP-PG4	rHEP-30V
1	3.31±0.15	5.50±0.14	5.75±0.18	5.77±0.22	6.06±0.15
2	4.28±0.31	6.69±0.20	7.28±0.16	7.25±0.05	7.62±0.14
3	4.43±0.08	7.10±0.17	7.89±0.10	7.91±0.10	8.13±0.08
4	4.35±0.25	7.42±0.22	7.99±0.12	8.12±0.11	8.12±0.03

Supplementary Table S7. Primers used for sequence analysis.

Primer	Forward primer (5'–3')	Position*	Reverse primer (5'–3')	Position*
RABV1	ACAGACAGCGTCAATTGCAAAGC	28–50	TTGACGAAGATCTTGCTCAT	1533–1514
RABV2	CTTCCGTTCACTAGGCTTGAGTGGG	934–958	GGRGGTGGAAGCCACARGTCATCG	2602–2579
RABV3	TGATCTATCAGTRGAGGCTGAGATCGC	2092–2118	CTGAAGAGACATGTCAGACCATAG	3056–3033
RABV4	ATGRCGATGACYTGTGGCTTCCACC	2575–2599	CCCATGTTCCATCCATAAGTCTAAG	4095–4071
RABV5	TATCCCGCAAGTTCATCACT	3113–3132	AGTTTGGCAGAGTCCTCAATC	5556–5536
RABV6	GGGTTTGGAAAAGCATATACCATATTC	4302–4328	GACTTGGAATAGAAATGGGCCAAGTC	5790–5765
RABV7	TGTCCCCAACATCTTGAGGAACTC	5488–5510	CGCATTGGTGGATACTGTAGA	7912–7892
RABV8	TACTAGCTCAAGGAGACAACCAGGT	7581–7605	TGAACCAGTTTATAGATTCTTTTAACG	9017–8991
RABV9	TCAGAGTTTCGAGAGGCAATCCTG	8399–8422	AGCTGCATGGCGCACCTCTTGATC	10249–10226
RABV10	CAGCTCAGGGGCTCTTATACTCAATC	9555–9580	CCAGAGGTTCGGATTCAAGA	11880–11861

*: The positions of the primer locations were defined based on the newly amplified genomic sequence of HEP using these primer sets.

Supplementary Table S8. Primers used for construction of infectious clones.

Primer	Forward primer (5'–3')	Position*	Reverse primer (5'–3')	Position*
Full-genome1	ACGCTTAACAACAAAACCAAAGAAG	1–25	TGAGCGATCTCAGCCTCYACTGATAG	2121–2096
Full-genome2	CTTCCGTTCACTAGGCTTGAGTGGG	934–958	GGACCAAGTTTGTCTGGTATCG	3412–3391
Full-genome3	CTATGGTCTGACATGTCTCTTCAG	3033–3056	GACTTGGAATAGAAATGGCCAAGTC	5790–5765
Full-genome4	TGTCCCCAACATCTTGAGGAACTC	5488–5511	CGCATTGGTGGATACTGTAGA	7912–7892
Full-genome5	TACTAGCTCAAGGAGACAACCAGGT	7581–7605	AGCTGCATGGCGCACCTCTTGATC	10249–10226
Full-genome6	CAGCTCAGGGGCTCTTATACTCAATC	9555–9580	ACGCTTAACAAATAAACAATAAAGAT	11925–11900
Kpn_HamRz_HEP	ATAGGTACCTGTTAAGCGTCTGATGAGTCCGTGAGGACGAAACTATAGGAAAGGAATTCCTATAGTCACGCTTAACAACAAAACCAAAGAAGAAGCA*	1–30		
Pst_HdvRz_HEP			CGGCTGCAGCGCCCTCCCTTAGCCATCCGAGTGGACGTGCGTCCTCCTTCGGATGCCCAGGTCGGACCGCGAGGAGGTGGAGATGCCATGCCGACCCACGCTTAACAAATAAACAATA*	11925–11905

Note that ribozyme sequences are underlined.

*: The positions of the primer locations were defined based on the newly amplified genomic sequence of HEP using these primer sets.

Supplementary Table S9. Primers used for construction of helper plasmids

Primer	Forward primer (5'–3')	Position*	Reverse primer (5'–3')	Position*
helper N	ATAGGTACCATGGATGCCGACAAG	67–85	CGGCTGCAGTTATGAGTCACTCG	1423–1410
helper P	ATAGGTACCATGAGCAAGATCTTTG	1511–1529	CGGCTGCAGTTAGCATGATGTGTAG	2408–2392
helper G	ATAGGTACCATGGTTCCTCAGGTTC	3318–3333	CGGCTGCAGTCACAGTCTGGTCTCG	4892–4877
helper L	ATAGGTACCATGCTGGATCCGGGA	5411–5425	CGGCTGCAGTTACAAACAACTGTAG	11794–11779

*: The positions of the primer locations were defined based on the newly amplified genomic sequence of HEP using these primer sets.

Supplemental Table S10. Primers used for construction of recombinant viruses.

Primer	Forward primer (5'–3')	Position*	Reverse primer (5'–3')	Position*
HEP-10V_P_L115H	AGATTCCACAAGATATGGTCACAG ACCGTAGAG	1850–1882	TATCTTGTGGAATCTCTCTCCTGACCT CATTTG	1864–1832
HEP-10V_G_S15R	CCCTGGAGACCTATTGACTTACACC ATCTCAGC	3411–3443	AATAGGTCTCCAGGGACCAAGTTTGT CTGGTAT	3425–3393
HEP-10V_L_L2055E	TGGTCCGAGGACACCCCAGTGTTC AAGAGGGTA	11567–11599	GGTGTCCTCGGACCAGCTCCAAGATA GATAGAT	11581–11549
HEP-30V_L_E753D	GAGTTAGATAGCATATCGAGGAAT GCACTCTCA	7661–7693	TATGCTATCTAACTCATAGAGAAGCC CCTCTTG	7675–7643
HEP-30V_G_V164E	ATAACGGAGTCCTCGACCTACTGC TCAACTAA	3858–3889	CGAGGACTCCGTTATTCCTGAGCAAT TTCCGCC	3872–3840
HEP-30V_G_L183P	GAGAATCCGAGACTAGGGACATCT TGTGACATT	3915–3947	TAGTCTCGGATTCTCAGGCATCCAGA TGGTGTA	3929–3897
HEP-30V_G_A286V	CTGGATGTACTAGAGTCCATCATG ACCACCAAG	4224–4256	CTCTAGTACATCCAGACACTCCTCTC TTTTCTTG	4238–4205

*: The positions of the primer locations were defined based on the newly amplified genomic sequence of HEP using these primer sets.

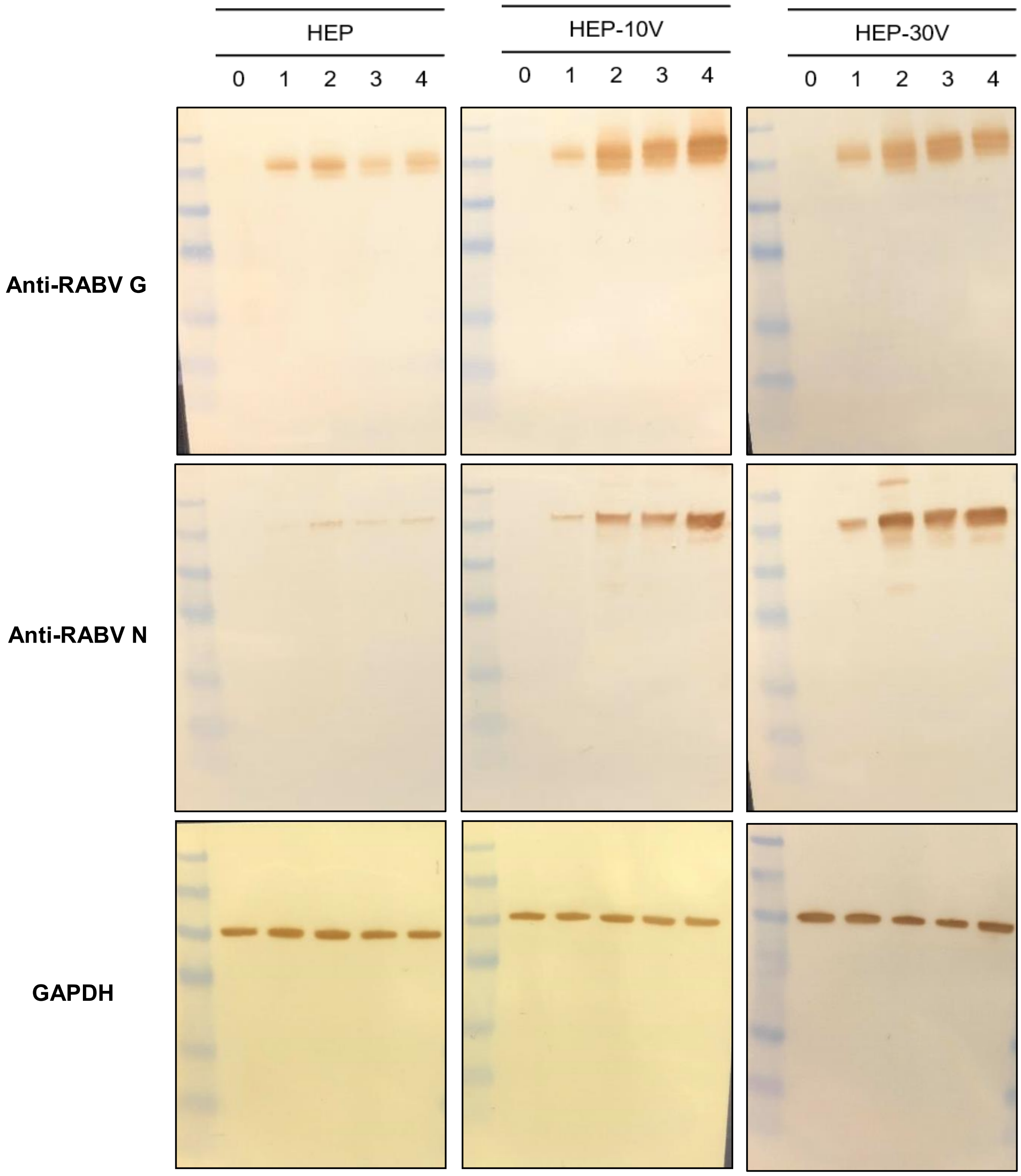

Supplemental Figure S1. Full uncropped Blot images for Fig.2(a)
